# Genomic distances reveal relationships of wild and cultivated beets

**DOI:** 10.1038/s41467-022-29676-9

**Published:** 2022-04-19

**Authors:** Felix L. Sandell, Nancy Stralis-Pavese, J. Mitchell McGrath, Britta Schulz, Heinz Himmelbauer, Juliane C. Dohm

**Affiliations:** 1 University of Natural Resources and Life Sciences, Vienna, Department of Biotechnology, Institute of Computational Biology, Vienna, Austria; 2 USDA-ARS, Sugarbeet and Bean Research Unit, East Lansing, MI USA; 3 KWS SAAT SE & Co. KGaA, Einbeck, Germany

**Keywords:** Classification and taxonomy, Phylogenomics, Plant breeding, Plant domestication

## Abstract

Cultivated beets (*Beta vulgari*s ssp. *vulgaris*), including sugar beet, rank among the most important crops. The wild ancestor of beet crops is the sea beet *Beta vulgaris* ssp. *maritima*. Species and subspecies of wild beets are readily crossable with cultivated beets and are thus available for crop improvement. To study genomic relationships in the genus *Beta*, we sequence and analyse 606 beet genomes, encompassing sugar beet, sea beet, *B. v. adanensis*, *B. macrocarpa*, and *B. patula*. We observe two genetically distinct groups of sea beets, one from the Atlantic coast and the other from the Mediterranean area. Genomic comparisons based on k-mers identify sea beets from Greece as the closest wild relatives of sugar beet, suggesting that domestication of the ancestors of sugar beet may be traced to this area. Our work provides comprehensive insight into the phylogeny of wild and cultivated beets and establishes a framework for classification of further accessions of unknown (sub-)species assignment.

## Abstract (web summary)

While a large amount of genomic resources is available, the phylogeny of wild and cultivated beets remains unclear. Here, the authors use the k-mer-based Mash method to analyze resequenced genomes of 606 accessions of the genus *Beta* and reveal Greece as the domestication site of sugar beet.

## Introduction

Beets are core eudicot plants of the genus *Beta* affiliated with the order of Caryophyllales, a taxonomic group that aside from beets includes crops such as spinach, quinoa, amaranth, buckwheat and *Opuntia*. A molecular taxonomy of Caryophyllales as well as their relationship to other major plant clades has been presented recently^1,2^.

According to the nomenclature of Ford-Lloyd & Williams^3^, the genus *Beta* is subdivided into the three sections *Beta* (formerly *Vulgares*), *Corollinae* and *Nanae*. A fourth section, *Procumbentes*, was described, but the single genus affiliated with it is now considered a distinct genus *Patellifolia*^4^. However, over the years, many changes to the taxonomical grouping of beets have been suggested^5^. The section *Beta* comprises all cultivated forms of *Beta vulgari*s ssp. *vulgaris* which include sugar, table, and leaf beets (chard), as well as the wild beets *Beta vulgaris* ssp. *maritima* (sea beet) and *B. vulgari*s ssp. *adanensis*. Additional species affiliated with the section *Beta* are *B. macrocarpa* and *B. patula*. Within the section *Beta*, subspecies and species are intercrossable. Thus, members of section *Beta* have received particular attention by beet breeders as sources for genetic improvement of stress tolerance of cultivated beets.

Many molecular resources are available for the genus *Beta*, in particular for sugar beet. Of central importance is an annotated reference genome sequence based on a double-haploid sugar beet genotype^1,6^ accessible at http://bvseq.boku.ac.at. Sugar beet is a diploid species with 2n = 18 chromosomes and has not undergone recent whole-genome duplication^7,8^. Estimates of the sugar beet genome size range between 731 Mbp^1^ and 758 Mbp^9^. Several sugar beet genomes in addition to the reference were assembled *de novo* as well^1,10^, the chard (*B. v. vulgaris* var. *cicla*) genome sequence has been published^11^, and also the assembled genomes of wild-derived beet accessions have become available^12,13^.

The ancestor of all beet crops is the sea beet (*B. v. maritima*), a plant that is native to the coasts from Morocco up to the North Sea and in the Mediterranean area. In an attempt to clarify the origin of domesticated beets, Andrello et al.^14^ genotyped about 1000 wild and cultivated *Beta* accessions for about 10,000 single-nucleotide variants using a microarray. The authors differentiated up to nine genetic groups, six of which contained at least a few cultivated beet accessions. It was not possible to assign a single geographic origin to sugar beet, because the majority of sugar beet accessions clustered together with wild beets from many different countries. Thus, at present, the origin of domesticated beets is unclear. Andrello et al.^15^ inspected the relatedness of wild beets affiliated with section *Beta* and recognized four clusters, i.e., *B. macrocarpa*, *B. v. adanensis*, *B. v. maritima* (Mediterranean and Asian origin), and *B. v. maritima* (originating from the Atlantic coast and from Northern Europe). Their work focused on statistical analysis of variant data but the phylogenetic relationships between the studied genotypes remained unresolved. In the work by Romeiras et al.^16^, a phylogenetic analysis using chloroplast and rRNA gene sequences was conducted on a number of wild beets collected in mainland Portugal and in Macaronesia (Madeira, Cabo Verde, Açores). While the authors observed clear separation of *B. macrocarpa* from other beets, the phylogenetic relationships between *B. v. vulgaris*, *B. v. maritima* and *B. patula* could not be clarified. Touzet et al.^17^ also used chloroplast and nuclear datasets for phylogenetic analysis of wild beets, including *B. v. maritima* accessions collected at multiple localities throughout their range of distribution, as well as *B. v. adanensis* and *B. macrocarpa*. Their results indicated clear divergence of the wild beet *B. macrocarpa* from the remainder of accessions tested. *B. v. adanensis* genotypes were placed in a separate subtree. Finer scale dissection of the genetic relationship of many of these accessions would be useful to address their crop improvement potentials. In summary, while several studies have been conducted to clarify the phylogenetic relationships among wild beet accessions and between wild and cultivated beets, the picture that has emerged is far from complete, mainly because either just a few markers were used for the analysis, or because array data rather than genome-wide sequencing data were analysed.

In this work, in order to assess the phylogeny of beets in a comprehensive way, we set out to perform whole-genome sequencing on a large number of public accessions of wild and cultivated beets with the aim of obtaining insight into beet phylogeny and domestication. Additionally, we include commercial breeding lines and lines from the sugar beet breeding program of the USDA to uncover their genomic relationship. The phylogenetic analysis of a large number of datasets is not trivial and requires substantial computational power and computing time when employing traditional alignment methods. We therefore test an existing k-mer-based method of pairwise distance calculation and apply this approach on low-coverage whole-genome sequencing data, followed by phylogenetic tree construction. Our results indicate that alignment-free genomic comparisons lead to valid conclusions and insights into beet phylogeny and breeding history.

## Results

### Selection of *Beta* accessions

We performed whole-genome sequencing of 606 *Beta* accessions comprising wild beets and sugar beet accessions. The majority of these accessions was selected from public ex situ germplasm collections, i.e., either from the National Genetic Resources Program of the United States Department of Agriculture (USDA), or from the gene bank of the Leibniz-Institut für Pflanzengenetik und Kulturpflanzenforschung (IPK) in Gatersleben, Germany. Focusing on publicly available accessions increases the usefulness of large-scale projects as seeds are readily available in order to raise plants for future analyses^18^. Further selection criteria were a balanced proportion of sugar beet and sea beet accessions, the inclusion of *Beta* species of unknown subspecies assignment, and representative sampling of wild beets according to their geographic range of occurrence with focus on the coastlines of Western and Southern Europe as well as the Eastern Mediterranean. In addition, we included 30 modern sugar beet lines which were provided by three different seed companies and 48 sugar beet accessions which represent germplasm of the USDA sugar beet breeding program at East Lansing (Michigan, USA). In total, our study encompassed 239 sea beet accessions, 285 sugar beet accessions, 17 accessions with unclear species/subspecies assignment (*Beta* sp.), 29 *B. v. adanensis* accessions, 33 *B. macrocarpa* accessions, and three *B. patula* accessions. All 606 accessions were sequenced by us with a genomic coverage between 4-fold and 7.5- fold (Table 1, Supplementary Data 1). Phenotypic information (disease resistances, geographic sampling coordinates) was collected from the public USDA and IPK databases, if available.

**Table 1:** Number of accessions per species analysed in this study.

	Databases^a^	Proposed assignment^b^
*Beta vulgaris* ssp. *vulgaris*	285	291
*Beta vulgaris* ssp. *maritima*	239	237
*Beta vulgaris* ssp. *adanensis*	29	45
*Beta macrocarpa*	33	30
*Beta patula*	3	3
*Beta* sp.	17	—
Sum	606	606

^a^Numbers according to taxonomic information provided by USDA/GRIN and IPK databases.

^b^Numbers after our suggested reclassification based on phylogenetic analysis.

Particularly noteworthy is the increase in numbers of *B. v*. *adanensis* accessions (four of which are less evident than the others, see Table 2).

### Applying Mash at different levels of genome coverage

In order to analyse the phylogenetic relationship and population structure of a large number of beet accessions we considered an existing k-mer-based method, Mash^19^, as most promising for low-coverage whole-genome sequencing data. This alignment-free method reduces sequencing data or assembled sequences to a selection (sketch) of representative subsequences (k-mers) of a chosen length k. Comparing k-mer sketches to obtain pairwise genomic distances is computationally cheap and sketch databases are small so that they can be easily shared with the research community. The discriminatory power of Mash had been demonstrated with high-coverage raw sequencing data of bacteria and with genome assemblies of primates^19^. To test the Mash approach with unassembled sequencing data of closely related complex plant genomes we generated Mash sketches from quality-filtered Illumina reads of two different *B. v. vulgaris* lines, two *B. v. maritima* accessions, two additional species of wild beets affiliated with the *Betoideae* subfamily, one member of the genus *Patellifolia* which is a close relative of *Beta*, and spinach (*Spinacia oleracea*) as an outgroup. The initial genomic coverage of the Illumina sequencing data was 20-fold for each sample. We calculated a phylogenetic tree based on Mash distances from pairwise comparisons of data sketches (see Methods).

**Fig. 1:**
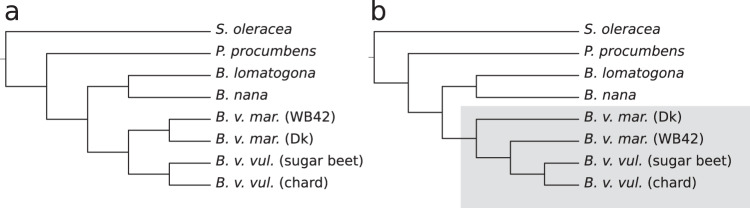
Phylogenetic trees from whole-genome sequencing data at different coverages based on Mash distances. Genomic coverages of the sequencing data before distance calculation were 20-fold, 5-fold, or 1-fold, respectively, all of which resulted in the same tree topology **a**. At coverage 0.5-fold **b** the topology of the phylogenetic tree partially changed (highlighted). Dk Denmark. Source data are provided as a Source Data file.

The resulting tree topology reflected the expected relationships between the species (Fig. 1a) in line with previous taxonomic studies^20^. Accessions from the genus *Beta* section *Beta* formed a monophyletic subtree with cultivated beets clustering together. *B. lomatogona* and *B. nana* which are members of two other sections of the genus *Beta* branched off separately, and the two species assigned to other genera (*Patellifolia* and *Spinacia*) were placed at the root of the tree.

To test whether Mash distances were reliably determined at lower coverages we randomly sub-sampled the sequencing data to 10-fold, 5-fold, 1-fold, 0.5-fold, and 0.2-fold genome coverage for each species and re-calculated pairwise distances and distance-based phylogenetic trees for each selection, keeping the sketch size constant. The topology of the calculated trees remained unchanged for a coverage as low as 1-fold. When decreasing the sequencing read coverage further, the subtree topology of *B. v. maritima* and *B. v. vulgaris* accessions was changed (Fig. 1b). Thus, we considered our average genomic coverage of 5.5-fold (±1.5) obtained for the 606 sequencing datasets from *Beta* accessions as sufficient for Mash analysis and phylogeny reconstruction.

We observed increasing Mash distances with each reduction of the genome coverage of the input data. The distance increase was larger for close nodes within the tree than for distant nodes. When lowering the coverage, the topology changed as soon as formerly close nodes became more distant to each other than to a formerly distant node. The explanation may be that at very low sequence coverage the genomes were no longer sufficiently represented so that the fraction of common k-mers was underestimated, resulting in artificially large distances. Since Mash distances of unassembled reads seem to be coverage dependent we concluded that it is advisable to use data sets with similar per-accession genome coverages to obtain reliable and comparable distance values for phylogeny reconstruction.

### Assessing the discriminatory power of Mash

**Fig. 2:**
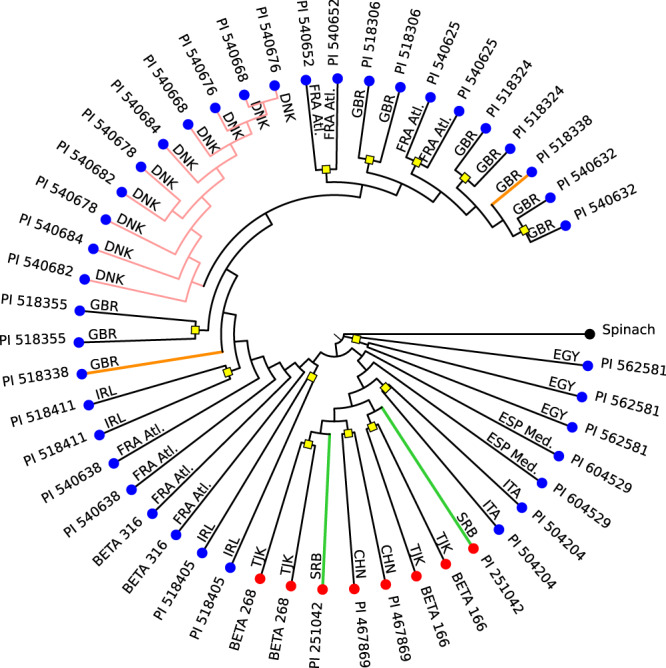
Phylogenetic tree of 23 *Beta* accessions and spinach as outgroup based on Mash distances calculated from whole-genome Illumina sequencing data. The 47 samples represent 23 *Beta* accessions. Pairwise clustering according to their accession identifiers is indicated by highlighting the respective nodes with a yellow square, discordant clustering is indicated by colored branches. Country designations are given at each branch, species designations are reflected in the leaf colors (red: *B. v. vulgaris*, blue: *B. v. maritima*, black: outgroup, Med.: Mediterranean, Atl: Atlantic). Source data are provided as a Source Data file.

The beet whole-genome sequencing data had been generated from very closely related genomes below the subspecies level. We therefore tested whether such close relationships could be resolved accurately by the Mash approach based on sequencing data of around 5-fold genomic coverage. To do so, we analysed 47 individuals from 23 beet accessions, i.e., 18 duplicates and one triplicate from *B. v. maritima* accessions and four duplicates from *B. v. vulgaris* (sugar beet) lines (taxonomic assignment according to databases at IPK and USDA/GRIN; accessions were maintained separately but had the same identifier). Geographic coordinates were available for all these accessions derived from seven European countries (Denmark, France, Ireland, Italy, Serbia, Spain, UK), two Asian countries (China, Tajikistan), and from Egypt. We asked whether Mash was able to (1) discriminate accessions of the same subspecies originating from different geographic locations and to (2) recognize samples derived from the same accession correctly (i.e., the expected tree topology should distinguish between wild and cultivated beets, cluster geographic subgroups together, and place duplicates or triplicates together in separate subtrees). Mash sketches were created, pairwise distances were calculated, and a phylogenetic tree based on Mash distances was generated from these samples including spinach as outgroup. In the resulting tree (Fig. 2), the four cultivated beet accessions (tips with red circles) formed a monophyletic group, showing that subspecies were correctly separated. The countries of origin clustered together in main groups of northern and southern countries with Egypt as the most distant subgroup. Most duplicated accessions and the triplicate were placed next to each other. Within the subtree of *B. v. maritima* accessions from Denmark, duplicates were not placed as pairs and, hence, had not been accurately resolved by the Mash approach, probably due to the close relationship of these accessions collected from sites being located only about 150 km apart from each other. We concluded that Mash would enable the separation of subspecies as well as regions of origin, though it might exhibit uncertain placements within groups of genetically and geographically very closely related accessions. The explanation would be that if genotypes are highly similar due to their geographic proximity, they share most of their unique k-mers, and Mash distances will be too small to resolve the topology unambiguously. Also, boundaries between accessions may be further confounded by heterozygosity as the sequenced accessions were not inbred, i.e., differences between individuals from the same accession may merely reflect their level of heterozygosity.

Removal of organellar DNA from the 47 samples described above resulted in a tree that was identical with Fig. 2, and we concluded that organellar DNA did not impact phylogeny reconstruction in our study (see Supplementary Notes 1). The explanation may be that organellar DNA is likely to be contained at high copy numbers^21^ and was not represented among the low-frequency k-mers that are used in the Mash analysis. Also, in plants, organellar DNA was described to be more conserved than nuclear DNA^22^.

As an additional test we analysed a subset of 255 *Beta* accessions using microarray-based genotyping and compared the resulting distance matrix and tree to the results based on Mash analysis using the same accessions (see Supplementary Notes 2, Supplementary Table 1). The two independent and very different approaches confirmed each other indicating that the Mash analysis was reliable.

Based on the various tests performed we concluded that for our *Beta* data (1) Mash analysis was reliable using whole-genome sequencing data with coverage as low as 5-fold, (2) discrimination of subspecies was successfully achieved, (3) accessions from similar regions of origin were correctly clustered together, (4) topologies may become uncertain for very closely related accessions or for very low coverages.

### Phylogenetic trees based on 474 *Beta* accessions

We initially assessed the phylogeny of the genus *Beta* based on whole-genome Illumina sequencing data from 474 beet samples (239 sea beet accessions, 218 sugar beet lines, and 17 accessions with unknown subspecies assignment) with a mean sequence coverage depth of 5.5-fold per accession before quality trimming. Trimming reduced the amount of data by about 5%. Mash was applied to extract k-mer sketches from each sequence dataset. Spinach and *P. procumbens* were included as outgroups, and pairwise distances for all accessions were determined using Mash.

**Fig. 3:**
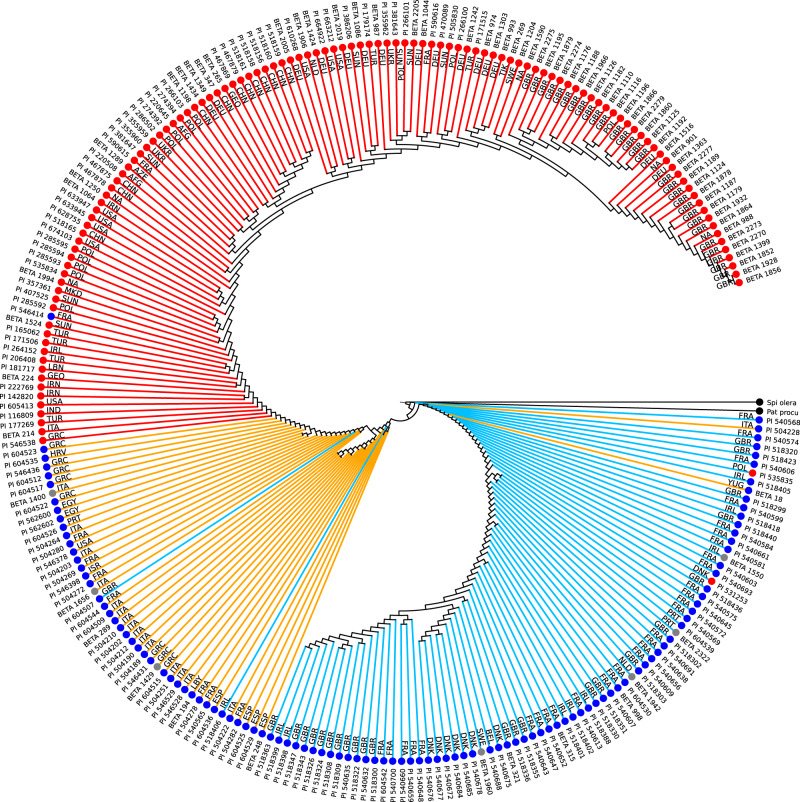
Phylogenetic tree of sugar beet and sea beet accessions based on Mash distances. The 457 randomly sorted input accessions were divided in two subsets one of which is shown in this figure, the other one in Supplementary Fig. 2. For comparability, 19 randomly selected accessions as well as the two outgroups spinach (Spi olera) and *Patellifolia procumbens* (Pat pro) are present in both trees. Tree calculation was performed with the Fitch algorithm based on pairwise Mash distances. Leaf colors indicate taxonomic information prior to tree calculation (red: *B. v. vulgaris*, blue: *B. v. maritima*, gray: unknown subspecies, black: outgroup). Branch colors indicate *B. v. vulgaris* (red), sea beets from the Mediterranean area (yellow), and sea beets from Atlantic coasts (blue). Country codes were added according to information in public databases from USDA or IPK. Source data are provided as a Source Data file.

The distance matrix of all pairwise distances was the basis for phylogenetic analyses. Several trees were calculated using the full matrix or subsets of accessions. As first observation we identified a group of 17 *B. v. maritima* accessions that showed a large distance to all other *B. v. maritima* accessions (Supplementary Fig. 1). These accessions were removed here and used again in a later analysis (see below). The resulting phylogeny using the remaining 457 accessions (Fig. 3, Supplementary Fig. 2, each of which showing about 50% of the input accessions for better readability) revealed an overall trend to form three large groups: one group comprising sugar beet accessions and two groups comprising sea beet accessions. The two sea beet groups were distinguished by their geographic location: one group originated from the Mediterranean area, the other one from Atlantic coasts. The trees showed that sugar beet accessions and Mediterranean sea beets were sister groups.

A few scattered accessions differed from the general trend and appeared within the group of the other subspecies. The phylogeny included accessions that were only labeled as *Beta* in the source database without subspecies assignment. Given the robustness of the Mash approach in terms of subspecies discrimination and geographical resolution our phylogeny provided evidence for 17 so-far unassigned *Beta* accessions as subspecies *vulgaris* or *maritima* according to their clustering patterns (Supplementary Fig. 4a and Table 2). Moreover, we may conclude that the few outliers of *B.v. maritima* within the *B.v. vulgaris* cluster (three accessions) and vice versa (four accessions) might be mislabeled entries in the public databases, as the tree topology shows their placement together with their genetically most closely related accessions of the other subspecies (Supplementary Fig. 4b and Table 2).

**Table 2:** Suggested taxonomic affiliation of beet accessions based on phylogenetic analysis.

Accession ID	Database assignment	Proposed assignment	Accession ID	Database assignment	Proposed assignment
BETA 1101	n.a.	MAR	PI 504206	MAR	ADA
BETA 1344	n.a.	MAR	PI 546437	MAR	ADA
BETA 1397	n.a.	MAR	PI 546439	MAR	ADA
BETA 1400	n.a.	MAR	PI 546441	MAR	ADA*
BETA 1429	n.a.	MAR	PI 546508	MAR	ADA
BETA 1550	n.a.	MAR	PI 546518	MAR	ADA
BETA 1656	n.a.	MAR	PI 546520	MAR	ADA
BETA 1762	n.a.	MAR	PI 546523	MAR	ADA
BETA 1942	n.a.	MAR	PI 562579	MAR	ADA
BETA 1960	n.a.	MAR	PI 562581	MAR	ADA
BETA 2322	n.a.	MAR	PI 562582	MAR	ADA
BETA 1214	n.a.	VUL	PI 562590	MAR	ADA
BETA 1228	n.a.	VUL	PI 562597	MAR	ADA
BETA 1320	n.a.	VUL	PI 604516	MAR	ADA
BETA 1773	n.a.	VUL	PI 604518	MAR	ADA*
BETA 2145	n.a.	VUL	PI 604545	MAR	ADA*
PI 502293	n.a.	VUL +	PI 518311	MAR	VUL
BETA 1693	ADA	MAR	PI 546414	MAR	VUL
BETA 1262	ADA	VUL	PI 604521	MAR	VUL
BETA 591	MAC	ADA*	PI 121838	VUL	MAR
BETA 6	MAC	MAR	PI 504178	VUL	MAR
BETA 7	MAC	MAR	PI 531253	VUL	MAR
BETA 2177	MAR	ADA*	PI 535835	VUL	MAR

ADA: *B. vulgaris* ssp. *adanensis*; MAC: *B. macrocarpa*; MAR: *B. vulgaris* ssp. *maritima*; VUL: *B. vulgaris* ssp. *vulgaris*; n.a.: not available. Asterisks indicate accessions at the root of the *B. v. adanansis* subtree, see Fig. 7. The plus sign indicates an accession described as fodder beet.

**Fig. 4:**
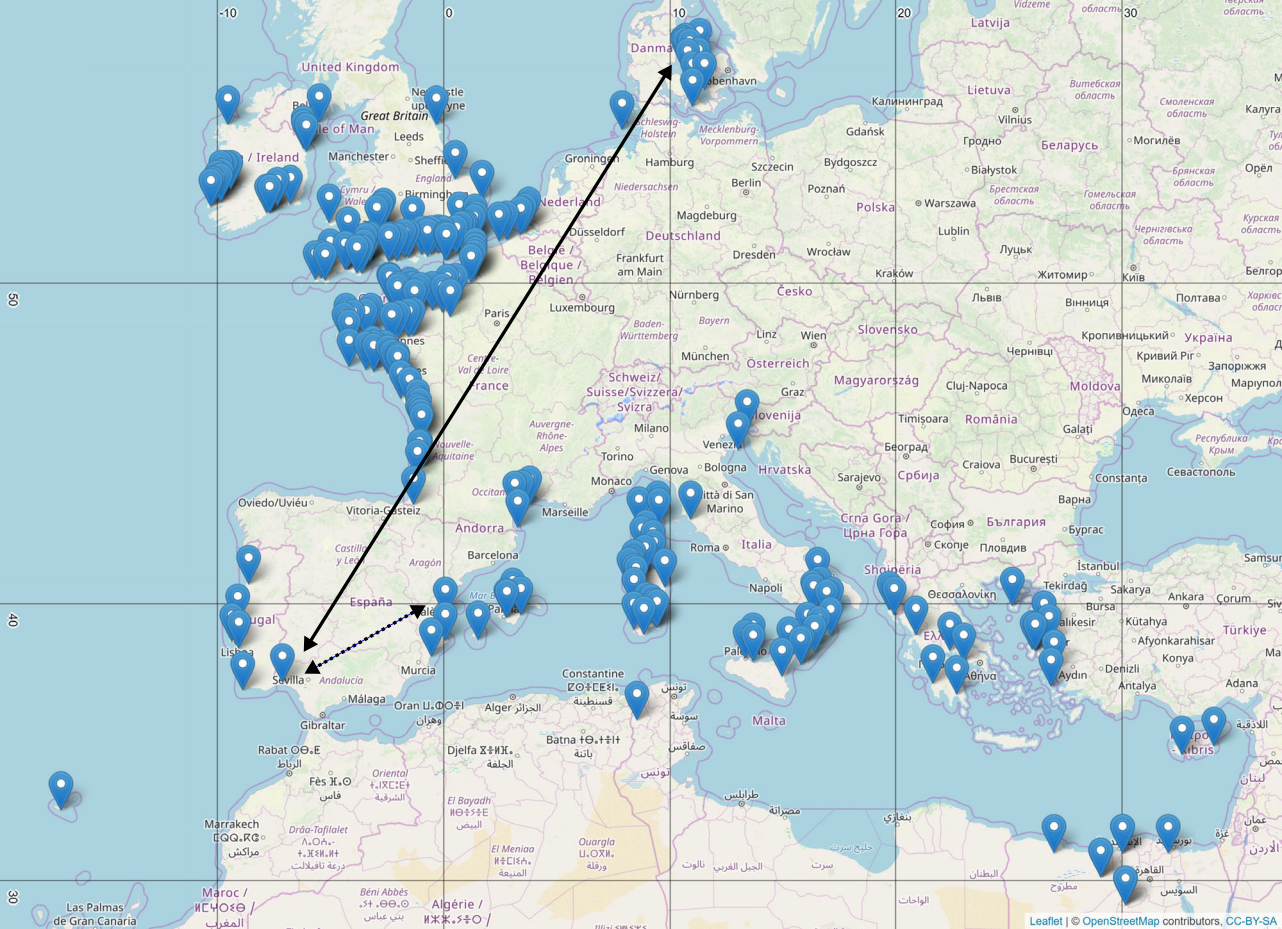
Location of sequenced wild beet accessions based on sampling coordinates obtained from public passport data. The black arrow points to two accessions separated by large geographical distance that show a small genetic distance. The dotted arrow points to accessions both originating from Spain and showing a larger genetic distance. The accessions are highlighted in the tree in Supplementary Fig. 3.

Our observation based on genomic sequencing data that Atlantic and Mediterranean sea beets form separate phylogenetic clusters confirmed a morphological study by Letschert et al.^23^ who concluded that “Oriental types” and “Atlantic types” of *B. v. maritima* could be distinguished. In our study, the genetic separation was most obvious for accessions originating from the Iberian Peninsula, depending on which side of the Strait of Gibraltar they had been collected from. For example, the Mash distance (0.0249) between sea beet accession PI 604540 originating from the Atlantic coast of Spain south-west to Seville and a sea beet accession from Denmark (PI 540673), separated by a geographic distance of about 2500 km, was smaller than the Mash distance (0.0257) between the Spanish accession PI 604540 and another Spanish accession from Valencia (PI 604528), i.e., derived from localities that are about only 700 km apart (Fig. 4, accessions highlighted in the tree in Supplementary Fig. 3). The separation indicates a barrier between the two sea beet populations in the region of the Strait of Gibraltar rather than continuous divergence along the European coasts coinciding with geographic distance. The assumption would be that wild beets originated in the Mediterranean region showing full diversity of the species and later expanded into northerly regions based on a smaller subpopulation.

Apart from a small number of sea beet accessions that were placed close to the root and could either appear in the Mediterranean subtree or in the Atlantic subtree there were two stable geographic outliers, i.e., BETA 18 described as originating from former Yugoslavia, which was placed within the Atlantic subtree next to Danish and Belgian accessions, and PI 604507 described as from Great Britain clustering with accessions from Italy and Spain. We cannot tell whether these accessions may have been mislabelled in the database, may be the result of human-mediated exchange, or may be an unexpected artifact of the phylogenetic analysis. We tend to exclude the latter option since the phylogenetic placement was repeatedly found in various tests (e.g., Supplementary Fig. 1).

In general, sea beets clustered together in subtrees according to their countries of origin and neighboring countries, outlining geographic regions of closely related accessions beyond country borders. Danish, Swedish, and Belgian sea beet accessions formed a subtree as well as British/French and British/Irish accessions indicating further substructuring of populations among the Atlantic *B. v. maritima* accessions.

One case of initially unexpected grouping concerned *B. v. maritima* accession PI 604526 labeled as originating from Portugal but appearing within the Mediterranean subtree and clustering together with accessions from Egypt. Closer inspection revealed that the geographic coordinates point to the Madeira archipelago located far south of the Strait of Gibraltar. This accession may be derived from plants that were introduced by humans to the island. Alternatively, the assumed barrier at the Strait of Gibraltar might affect only genetic exchange between Mediterranean and the northern Atlantic subpopulations and does not prevent the exchange along the African coast and its offshore Atlantic islands (Macaronesia). A previous study confirmed a major genetic distance between *B. v. maritima* subpopulations from the Spanish/Portuguese Atlantic coast and the Moroccan coast but also suggested that samples from Madeira were more closely related to the Spanish/Portuguese subpopulation than to the Moroccan subpopulation^24^. Sampling and analysing more accessions from Morocco, Spain, Portugal, and Macaronesia may shed light on the population structure of sea beets native to this region and its relation to the Mediterranean subpopulations.

### Origin of the sugar beet

The provenance of the *B. v. maritima* accessions that were placed as a sister group to the *B. v. vulgaris* subtree (Fig. 3, Supplementary Fig. 2) provided evidence for the area where sugar beet cultivation had presumably been initiated. Phylogenetic placement unequivocally showed that sugar beet accessions were genetically closer to Mediterranean sea beet accessions than to wild beets originating from the Atlantic coast. The sugar beet accessions in the phylogenetic tree were most closely related to *B. v. maritima* accessions from Greece which themselves were closely related to a cluster of wild beets of Egyptian origin. Thus, the phylogeny based on Mash distances of the sea beet accessions analysed in this study may suggest a central role of Greece as the place where the ancestors of sugar beet were domesticated. Previous studies (Frese et al.^25^ and references therein) assumed the earliest domestication of beets in the Middle East with further domestication in Turkey and Greece and introduction to Northern Europe from there. Here, we present genomic evidence that supports Greek wild beets as ancestors of sugar beets. Analysis of additional sea beet accessions from this area may further narrow down the region of origin.

### Phylogeny of sugar beet

**Fig. 5:**
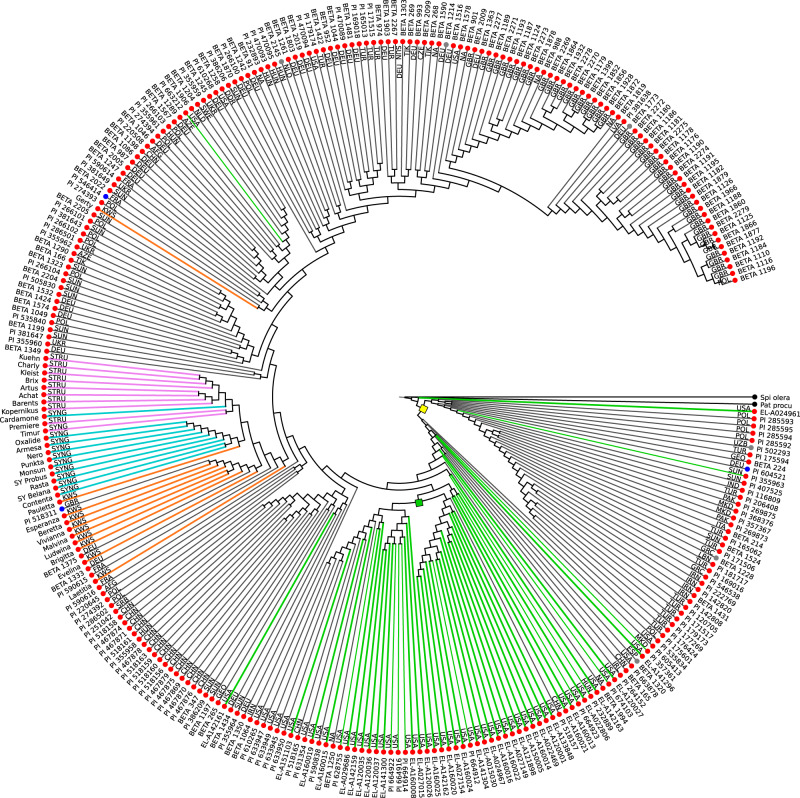
Phylogenetic tree based on Mash distances for 290 accessions identified as *B. v. vulgaris*. Leaf colors indicate the subspecies assignment in databases (red: *B. v. vulgaris*, blue: *B. v. maritima*, gray: no assignment, black: outgroup), branch colors indicate breeding programs (green: East Lansing, orange: KWS SAAT SE, cyan: Syngenta, magenta: Strube Research, black: none or unknown). Two nodes were highlighted by a yellow or green square, respectively (see text). Accessions mentioned in the text are highlighted in Supplementary Fig. 5. Source data are provided as a Source Data file.

An extended set of sugar beet accessions was used in a separate phylogenetic analysis without wild beet accessions. Accessions from three different seed companies were included (KWS SAAT SE, Strube D&S GmbH, Syngenta) representing current commercial breeding material. Together with additional 48 accessions from the academic sugar beet breeding program of the USDA at East Lansing (Michigan, USA) we calculated a phylogenetic tree for a total of 290 accessions from 28 countries (Fig. 5, Supplementary Fig. 5, Supplementary Notes 3). Several *B. v. vulgaris* subtrees showed a clustering by country of origin. As a subtree close to the root (the subtree to the right of the node marked in yellow) we found a broad geographic stretch from Europe to Iran, consistent with a broader sugar beet distribution prior to commercial activity and variety formation. Closer inspection revealed that a few of these accessions may be cultivated beets other than sugar beet (see Supplementary Notes).

Interestingly, three subtrees were formed by the cultivars obtained from each of the three breeding companies. The Mash approach allowed a detailed resolution of these very closely related sugar beet accessions so that on the one hand current breeding material may be distinguished from other publicly available accessions and on the other hand subtrees were distinguishable by seed company.

The accessions obtained from the East Lansing breeding program clustered together in the phylogenetic tree, reflecting their close relationship and showing a close relationship to further accessions from the USA in the same cluster. The East Lansing breeding lines are a mixture of populations under selection and official publicly available germplasm releases (indicated by PI numbers).

Most of the East Lansing entries fell within three subtrees (Fig. 5, the two relevant nodes marked in yellow or green, respectively), which included other USDA material as well, with the exception of breeding line NNS (EL-A142161; highlighted in Supplementary Fig. 5). The exceptional NNS is a germplasm making a move towards greater sugar-ness since most of its parents were elite agronomic performers of the East Lansing program. The subtree to the left of the node marked in green seems to carry more US Western releases than Eastern (subtree to the right of the node marked in green), and this might be expected from the major selection divergence of Western via resistance to Curly Top virus and Eastern to *Cercospora* leaf spot which took place in the 1930’s^26^. Background information and details on selected accessions (highlighted in Supplementary Fig. 5) are described in the Supplementary Notes.

In summary, the phylogenetic trees (Fig. 3, Supplementary Fig. 2 and Fig. 5) represent a comprehensive set of *B.v. maritima* and *B.v. vulgaris* accessions supporting our key conclusions: (1) two major populations of sea beets, one Mediterranean and one Atlantic population, can be distinguished and there seems to be limited genetic exchange between them, (2) sugar beets are closely related to the group of Mediterranean sea beets, (3) sugar beets show the lowest genetic distance to sea beet accessions collected in Greece, (4) sugar beet breeding history is reflected in their genomes so that they cluster by breeding programs and countries, and (5) a group of accessions close to the root of the sugar beet tree may represent a genomic state prior to commercial varietal diversification.

### Phylogeny of Mediterranean wild beets

**Fig. 6:**
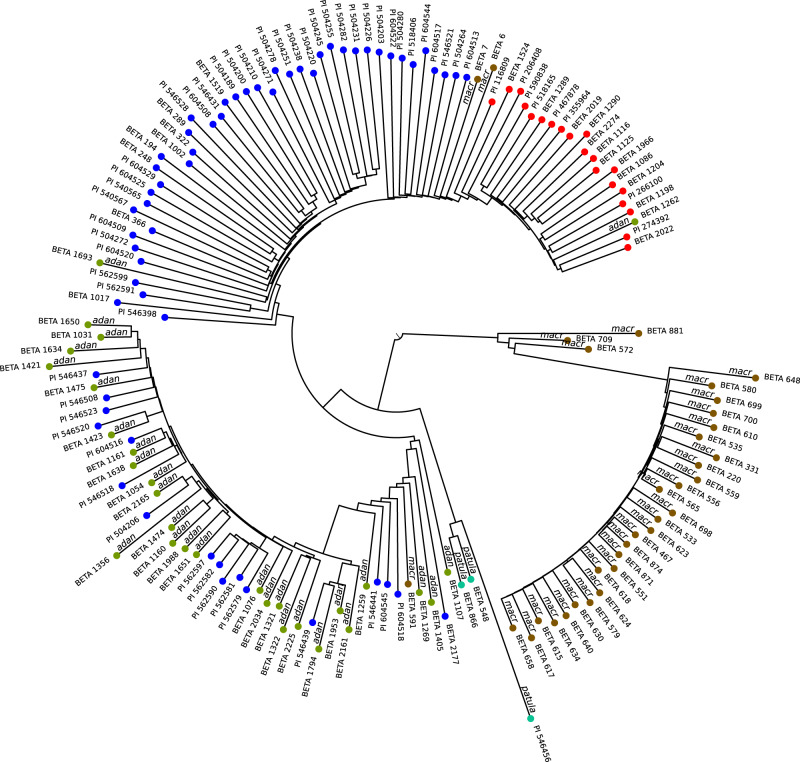
Phylogenetic relation of wild beet accessions based on Mash distances. A set of 60 sea beets from the Mediterranean area and 20 sugar beets (randomly selected) was analysed together with accessions of three further wild beet species. Node colors indicate taxonomic affiliation according to passport data. Blue: *B. v. maritima*, red: *B. v. vulgaris*, olive: *Beta vulgaris* ssp. *adanensis*, brown: *Beta macrocarpa*, turquoise: *Beta patula*. Source data are provided as a Source Data file.

The Mediterranean sea beet *B. v. maritima* co-occurs with another subspecies *B. v. adanensis*, as well as with *B. macrocarpa*, both of which are endemic to the Mediterranean area^15,27^. Another wild beet species of the *Beta* section is *Beta patula* endemic to the Madeira archipelago^13,28^. We sequenced accessions identified as *B. macrocarpa* (33 accessions), *B. v. adanensis* (29 accessions), or *B. patula* (3 accessions), respectively, according to their passport data available from the IPK database. After calculating pairwise Mash-distances we generated a phylogenetic tree including these wild beet species together with *B.v. maritima* accessions originating from the Mediterranean coast and together with a number of sugar beet accessions (Fig. 6). The resulting tree revealed separate subtrees for *B. macrocarpa* and *B. patula*. Another subtree contained the vast majority of *B. v. adanensis* accessions and was intermixed with accessions that had been labeled as *B. v. maritima* in the databases. For the same *B. v. maritima* accessions of this subtree we noted in initial analyses of only sea beets and sugar beets (see above) an exceptionally large distance compared to all other sea beet accessions (Supplementary Fig. 1). The observation that these accessions were now placed in a subtree closely interleaved with *B. v. adanensis* suggests that they are actually to be considered as *B. v. adanensis*. This result based on genomic distances using sequencing data supports previous findings based on single-nucleotide polymorphism (SNP) arrays^14^.

Accessions in the *B. v. adanensis* subtree show similar genetic distances to each other and are separated from *B. v. maritima* accessions with a distance as large as the distances between *B. macrocarpa* or *B. patula* accessions to *B. v. maritima*. Sugar beet accessions, however, form their own subtree within the subtree of sea beets, and only a small genetic distance separates these two groups that are both classified as *B. vulgaris* subspecies. The traditional classification of the cultivated form as a sister subspecies in relation to the wild species may be arbitrary. However, given the genetic distances as determined here between sea beet and sugar beet on the one hand and sea beet and other wild beets on the other hand it may be justified to classify *B. v. adanensis* as a separate species rather than a subspecies of *B. vulgaris*. The nine accessions that were placed at the root of the *B. v. adanensis* subtree showing a mixture of accessions labeled as *B. v. adanensis*, *B. macrocarpa*, and *B. v. maritima* may be interesting targets for further research as they might represent examples of genetic exchange between these three taxa. For now, we would consider these accessions as *B. v. adanensis* rather than *B. macrocarpa* or *B. v. maritima* according to their phylogenetic position (Table 2).

### Integration of the *Beta* phylogeny with phenotyping data

**Fig. 7:**
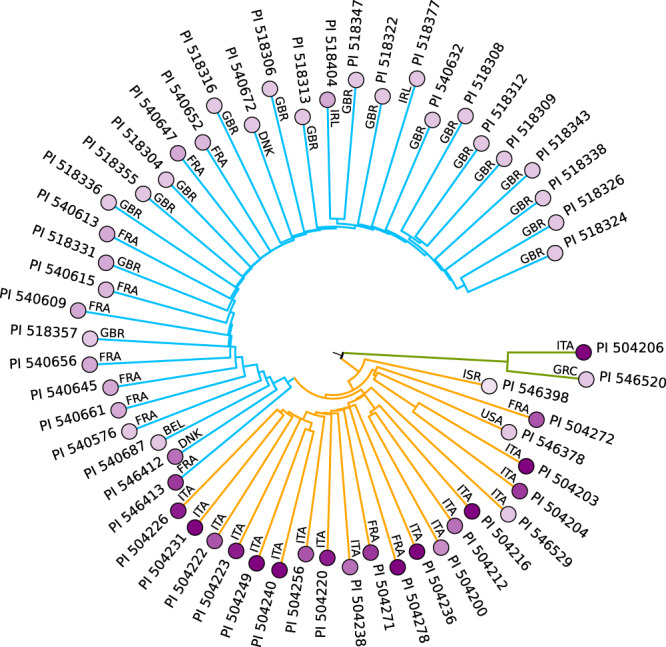
Phylogenetic tree of accessions used in studies on *Erysiphe* resistance and available in the USDA/GRIN database. Branch colors refer to sea beets from the Atlantic coast (blue), from the Mediterranean coast (yellow), and to sea beets that we would classify as *B. v. adanensis* (green). Color gradient of nodes reflects the resistance classification from light (susceptible) to deep purple (resistant) according to passport data. Countries of origin according to passport data are indicated.

The Mash distances and phylogenies we determined for the 606 different *Beta* accessions can be employed to quickly inspect the phylogenetic placement of accessions with known phenotypic properties, e.g., for the purpose of revealing closely related accessions that may have similar properties. We extracted information on disease resistances from the USDA/GRIN database (https://www.ars-grin.gov/) and selected the resistance against the mildew *Erysiphe betae* as one example for the integration of phenotypic information with the *Beta* phylogeny. Instead of calculating a new tree we extracted the branches of 56 accessions with *Erysiphe* resistance information from an initial tree containing all sea beet accessions and displayed the resistance strength per accession as color gradient (Fig. 7). The resulting subset of the tree revealed that, in general, Mediterranean sea beets were resistant and Atlantic sea beets were susceptible. Since *Erysiphe betae* was described as being most damaging in warm and arid climates^29^ the resistance was indeed expected to be found in Mediterranean areas rather than in northern sea beet habitats. However, some Mediterranean accessions show stronger resistance than others which may be of interest for the selection of yet unstudied accessions being placed as neighbors in the comprehensive tree. Interestingly, two accessions that we would classify as *B. v. adanensis* based on their large distance to other sea beets (see above) were included in *Erysiphe* resistance studies, and one of them (PI 504206) was found to be resistant whereas the other one (PI 546520) was identified as susceptible (Fig. 7, green branches). Such pairs of accessions of close genetic distance and different phenotypic properties may be interesting targets for comparative studies. Also, given the large phylogenetic distance between *B. v. maritima* and *B. v. adanensis*, the genetics behind the resistances may differ between these taxa as resistance may be promoted by alleles of the same gene, or alternatively, entirely different genes may be involved.

## Discussion

We provide insight into the phylogenetic relationship of wild and cultivated beets based on whole-genome sequencing. A random selection of sequencing reads from each sample was compared using subsequences (k-mers) of the reads and resulted in pairwise genetic distances for a large number of accessions of the genus *Beta*. Extensive testing was conducted to verify the usefulness and accuracy of a k-mer-based approach^19^ for the phylogenetic analysis of closely related plant genomes sequenced at low coverage. We showed that distance calculation mediated by a k-mer-based method on sequencing data achieves highly similar results when compared to the more traditional method of microarray-based genotyping. We were able to resolve the genetic relationship of 606 *Beta* accessions comprising 285 sugar beet accessions and 321 wild beet accessions to greater detail than previous studies.

Sea beets formed two large groups related to their Mediterranean or Atlantic origin, respectively, and sugar beets clustered together as a sister group to Mediterranean sea beets. Sea beets from Greece were the ones showing the closest genetic distance to cultivated beets. The separation of sea beet accessions originating from Atlantic or Mediterranean coasts seems to be triggered by a genetic barrier coinciding with the Strait of Gibraltar. Since the propagation of sea beets relies on seed dispersal through water^30,31^ there may be a physical barrier due to ocean currents. Oceanographic barriers have been described as a reason for gene flow reduction between species, and the Strait of Gibraltar was identified as one of them^32^.

In our phylogeny, the wild beets *B. macrocarpa*, *B. patula*, and *B. v. adanensis* formed their own monophyletic subtrees and revealed a number of *B. v. maritima* accessions that seem to be mislabelled in the seed databases from which they were obtained. Whether *B. v. adanensis* should be treated as subspecies of *B. vulgaris*, or as a separate species may be considered. Several scattered outliers in all major subtrees point to further misclassified accessions and were compiled in a table provided with this article (Table 2, Supplementary Data 1). For *Beta* accessions of unknown subspecies included in our analysis we propose a subspecies assignment based on the placement in our phylogenetic trees. These findings testify to the importance of supporting taxonomic classification of wild beets with molecular data, especially when dealing with accessions originating from areas where several species or subspecies of wild beets co-occur and morphological distinction may be difficult^33^. Our approach may be applied to further accessions from ex situ collections in order to determine or verify their origin or (sub-)species assignment by using the framework of the phylogeny established by us. The accessions analysed were obtained from public seed repositories, i.e., our findings are directly usable with this publicly available seed material. Based on our phylogeny one can select accessions of exceptionally close or distant relationship for further studies on species of genus *Beta*, section *Beta*. Furthermore, our study may serve as an example for analysing the population structures of other species.

Since the k-mer-based approach determines one single value as distance per pair of accessions, direct conclusions on specific genomic regions responsible for the divergence between accessions will not be possible. However, once accessions of interest are identified using such distances, the sequencing data generated may be used for mapping against a reference to find specific differences in the genomes. Alternatively, the accessions may be sequenced at deeper coverage to apply SNP-based approaches.

The discriminatory power of the k-mer-based approach was demonstrated in an analysis comprising a large number of solely sugar beets (Fig. 5). Although their distances were extremely small (average distance: 0.0254), a striking feature of the phylogenetic analysis was the apparent separation of sugar beet accessions by location. Beets used for animal feed (fodder beets) predate their use for sucrose production, and this use of *B. vulgaris* as fodder was likely widely dispersed across Europe. Historical evidence suggests that sugar beet was derived from Silesian fodder beets by the breeding efforts of F. C. Achard at the end of the 18th century, following A. S. Marggraf’s discovery of beet sugar as sucrose^34^. The subtree close to the root containing accessions from several countries may represent such material before commercial sugar beet breeding activity. The observed relatedness within each company’s germplasm may reflect the effect of inbreeding from different progenitors from an originally heterogeneous pool and perhaps bona fide parents in common, e.g., same seed parent but different pollen parents or vice versa. The phylogenetic tree is consistent with a scenario whereby sugar beet breeding followed a path of assortment from an ancestral population rather than a step-wise evolution^35^. Thus, it might be suggested from our data that early sugar beet lines were extracted from the entire range at one time or another, with the surviving commercial sugar beet materials being simultaneously developed in Central and Western Europe from extant fodder beets^36^ prior to their diversification showing strong relatedness within each breeding company.

All commercial accessions tested here were hybrids facilitated by a complex system of cytoplasmic male sterility (CMS) and restorer genes^37^. Typically, CMS seed parents are subject to intensive selection for at least two recessive fertility restoration genes as well the required recessive monogerm trait which allows beets to be planted for optimal field density and reduced cultivation labor. Thus, k-mer analyses of these commercial hybrids reflect the contributions of the CMS seed parent from intensive selection for at least three recessive genes. As CMS only became widely used from the mid-1970’s onwards, and was derived or inspired from the original development and deployment of CMS parents by the USDA, it is likely in part that the commercial provenance observed reflects proprietary extractions and performance optimizations of CMS seed parents from these progenitor materials; each company started with the same limited set of CMS seed parents and restorer lines and have since diverged in isolation by seed company (e.g., founder effects). In contrast, pollen parents generally have been selected for different traits than CMS seed parents, for mostly dominant and additive characters contributing to the profitability of the crop such as location-specific disease and stress resistances and general sucrose accumulation traits. Diversification within each company could reasonably reflect, in part, development of hybrids for their specific markets niche.

The integration of phenotypic data with the *Beta* phylogeny may facilitate the selection of accessions for breeding activities. For example, resistances against a particular stressor can arise multiple times, so that different genes or different alleles of a particular gene may be involved in disease resistance. Knowledge about the phylogenetic position of resistant genotypes may guide and direct further breeding efforts. Possible scenarios could encompass a) screens of closely related wild beets for disease resistance in order to discover resistant or susceptible accessions, b) discovery of new resistances by phenotyping clades that currently lack resistance information, c) exclusion of accessions from testing because their phylogenetic grouping suggests presence of resistance genes or alleles already recorded in other closely related accessions.

In summary, our study contributes valuable insights and comprehensive resources related to the genetic relationship between accessions of sugar beet and its wild relatives of the genus *Beta*. Future research may complement our findings with the analysis of additional accessions from regions of the Middle East or North Africa that were not widely covered yet.

## Methods

### Plant material, DNA isolation, and genotyping

Seeds from a total of 606 *Beta* accessions were obtained from the public germplasm repositories of the USDA (https://www.ars-grin.gov/) and the IPK (https://www.ipk-gatersleben.de/), from three sugar beet breeding companies (KWS SAAT SE, Syngenta, Strube Research GmbH), and from the USDA sugar beet breeding program at Michigan State University, East Lansing, MI, USA. All plants were grown in the greenhouse at KWS, and leaf material was harvested from one single plant per accession approximately six weeks after sowing. Plant genomic DNA for genotyping and sequencing was extracted by a silica-membrane method using the NucleoSpin 96 Plant II kit (Macherey-Nagel, Düren, Germany). Genotyping data were produced with the Illumina Infinium HD-chip workflow according to the manufacturer´s instructions (Illumina, San Diego, CA, USA). Following quantification of genomic DNA by Qubit fluorometry (Life Technologies, Foster City, CA, USA), 200 ng of each DNA sample was sheared to a peak size of 580 bp with a Covaris M220 instrument (Covaris, Woburn, MA, USA).

### Genomic sequencing and data processing

Sequencing libraries were prepared with the TruSeq Nano LT library preparation kit (Illumina; kit numbers FC-121-4001 and FC-121-4002) using indexed adapters. Quality and quantity of the libraries was assessed using a DNA 1000 chip on the Bioanalyzer 2100 instrument (Agilent, Santa Clara, CA, USA). Sixteen libraries with different indices were pooled into a sequencing lane, aiming at 5-fold genome coverage for each sequenced sample. Paired-end sequencing with 2 × 125 nt read length was performed on an Illumina HiSeq2500 instrument using v4 sequencing chemistry. Sequencing data were demultiplexed. Assuming the beet genome size at 758 Mbp, we calculated the genomic raw read coverage for each sequenced accession. Aiming at a genomic coverage of 5-fold, accessions with less genomic coverage were sequenced again from the same libraries in another sequencing run, and data from both runs were pooled. Datasets with coverage above 7-fold were downsampled to a coverage of 5.5-fold using seqtk v1.0-r82-dirty (seqtk sample [fraction]) (https://github.com/lh3/seqtk). Quality filtering and sequencing read trimming was done using trimmomatic^38^ v0.35 with the following settings: LEADING: 28 TRAILING:28 SLIDINGWINDOW: 5:15 MINLEN:50. Tests of the suitability of Mash for the analysis of low-coverage sequencing data included published data from sugar beet accession KWS2320 and *Spinacia oleracea* (spinach) Viroflay^1^, *B. v. maritima* accession WB42^12^, chard M4021^11^, as well as custom-prepared data from *Patellifolia procumbens* (BETA 951), *Beta nana* (BETA 541), *Beta lomatogona* (BETA 825), and *B. v. maritima* accession DkYBm. Accessions with BETA identifiers were obtained from IPK, Gatersleben, Germany. DkYBm was obtained from Syngenta. Both WB42 and DkYBm originate from Denmark. For testing the impact of organellar DNA we removed organellar DNA by mapping quality filtered sequencing reads against the genomes of the sugar beet chloroplast^39^ and mitochondrion^40^ using bowtie2^41^ v2.2.6 keeping only the non-matching reads (parameters -p 30, -X 1000, --un-conc).

### Generating data sketches

Sketches were generated using the *sketch* function of Mash^19^ v2.0. With the *sketch* function (parameters -k 21, -s 10000, -m 2), a set of sequencing reads was converted into a MinHash sketch, ignoring single copy k-mers. For benchmarking Mash performance on highly similar genomes, sequencing datasets with coverage between 30-fold and 100-fold were used. Bacteriophage PhiX sequencing reads contained in the dataset were removed using bowtie2 (parameters -p 20 -X 500). Thereafter, data downsampling to coverages from 0.2-fold to 20-fold, sequencing read quality trimming, and sketch calculation was performed.

### Distance matrix calculation and phylogenetic analysis

SNP data from the Infinium HD-chip were reduced to 19373 by eliminating non-functional assays and monomorphic markers. Pairwise genetic distances were calculated as Rogers’ genetic distance (RD)^42^ considering the absence of a SNP marker as a missing value. RD calculations were performed using the SelectionTools package v15.1.1 (http://population-genetics.uni-giessen.de/~software/^43^) implemented in R^44^. For sequence-based clustering, pairwise sketch distances were calculated employing the *dist* function implemented in the Mash program. Distance matrices were compared using the Mantel test^45^ as implemented in the R package vegan^46^ v2.5.7 (R v4.0.4). The Pearson correlation coefficient with 999 permutations was used as correlation method. Phylogenetic trees were calculated using the Fitch algorithm^47^ included in PHYLIP^48^ v3.696. We randomized the input order in each tree and used spinach and *P. procumbens* as outgroups. Trees were generated using five times jumbling (trees from 457 sea beets and sugar beets), ten times jumbling (tree of sugar beet accessions only; distance tree of 474 beet accessions), five times jumbling and global rearrangements (wild beet tree), or ten times jumbling and global rearrangements (trees to test the Mash approach), respectively. Phylogenetic trees were visualized using the Newick utilities toolkit^49^ v1.6. Tree topology comparisons were achieved by calculation of quartet distances^50^ of phylogenetic trees using the program quartet_dist of the software package tqDist^51^ v1.0.2 with standard parameters (quartet_dist -v).

### Annotation of phylogenetic trees

Accessions were labeled in phylogenetic trees according to their passport data in public databases or according to the breeding program or company. The information for most accessions includes species/subspecies assignment, country of origin, resistance information, and geographic coordinates that were obtained from the databases of USDA/GRIN (https://www.ars-grin.gov/) and IPK (https://www.ipk-gatersleben.de/gbisipk-gaterslebendegbis-i/).

### Geographic maps

Geographical maps showing sample locations and disease resistances were created using the open source JavaScript library leaflet v2.0.4.1 (https://leafletjs.com/) and OpenStreetMap licensed under the Open Data Commons Open Database License (https://opendatacommons.org/licenses/odbl/) by the OpenStreetMap Foundation (https://osmfoundation.org/).

### Computing resources and scripting

Programs and commands were run on a Linux computing cluster featuring a CentOS 6.7 operating system with seven computing nodes each equipped with either 24 (3.3 Ghz) or 32 (2.8 Ghz) cores. An average of 128 GB of RAM was used throughout the analysis. Scripts to automate analysis steps were coded with bash v5.0 and Perl v5.10.1.

### Reporting summary

Further information on research design is available in the Nature Research Reporting Summary linked to this article.

### Supplementary information

Supplementary Information

Description of Additional Supplementary Files

Supplementary Data 1

Reporting Summary

### Source data

Source Data

## Data Availability

Sequencing data generated in this study have been deposited in the NCBI SRA database under BioProject PRJNA815240 with accession numbers SAMN26581974-SAMN26582602. K-mer sketches^52^ [10.6084/m9.figshare.19222410] and Mash distances^53^ [10.6084/m9.figshare.19292054] generated in this study are available at FigShare. Source files for geographic maps^54^ generated based on coordinates (obtained from USDA/GRIN and IPK databases) and resistance information (obtained from USDA/GRIN database) have been deposited at FigShare [10.6084/m9.figshare.19222512]. These maps showing the distribution of sequenced sea beet accessions and selected resistances are available at https://bvseq.boku.ac.at/geoMaps/. Source data are provided with this paper.
