# Author Correction: Genomic distances reveal relationships of wild and cultivated beets

**DOI:** 10.1038/s41467-024-45401-0

**Published:** 2024-02-05

**Authors:** Felix L. Sandell, Nancy Stralis-Pavese, J. Mitchell McGrath, Britta Schulz, Heinz Himmelbauer, Juliane C. Dohm

**Affiliations:** 1 University of Natural Resources and Life Sciences, Vienna, Department of Biotechnology, Institute of Computational Biology, Vienna, Austria; 2 USDA-ARS, Sugarbeet and Bean Research Unit, East Lansing, MI USA; 3 KWS SAAT SE & Co. KGaA, Einbeck, Germany

**Keywords:** Classification and taxonomy, Phylogenomics, Plant breeding, Plant domestication

Correction to: *Nature Communications* 10.1038/s41467-022-29676-9, published online 19 April 2022

Since the publication of this work, Felix L. Sandell has changed their name from Felix L. Wascher. This has now been amended in the HTML and PDF versions of the article.

