# Supplementary material for: Genomic distances reveal relationships of wild and cultivated beets
Source: Nat Commun. 2022 Apr 19;13:2021. doi: 10.1038/s41467-022-29676-9 (PMC9019029; doi:10.1038/s41467-022-29676-9)
Supplement: Supplementary file 1 — Supplementary Information [file 41467_2022_29676_MOESM1_ESM.pdf]

Genomic distances reveal relationships of wild and cultivated beets

Wascher *et al*.

Supplementary Note 1. Testing the impact of organellar DNA

We evaluated if the presence of organellar sequences in the data had an impact on phylogeny calculation. We removed organelle-derived sequencing reads from the set of 47 samples (described in paragraph "Assessing the discriminatory power of Mash" of the main text) by mapping against the genomes of the sugar beet chloroplast[1] and mitochondrion[2], respectively. Reads not matching organellar genomes (about 93%) were used to create k-mer sketches based on which we determined Mash distances for tree calculation. The correlation between the distance matrices before and after removal of organelle-derived reads was r=1 (Mantel test[3]) and the resulting tree was identical with the tree in Fig. 2. We concluded that organellar DNA did not impact our phylogenies. The representative genome sketches generated by Mash that are used for distance calculation comprise the 10,000 least-frequent 21-mers of the input data (omitting single-copy k-mers). We can expect that k-mers from organellar DNA are contained in high copy number in the sequencing data and therefore not represented in the sketches.

Supplementary Note 2. Comparison of genetic distances obtained from two approaches

We compared distances calculated from single-nucleotide polymorphisms assessed by microarray-based genotyping to Mash distances, using a subset of 255 *B. v. maritima* accessions. To avoid intra-accession heterogeneity, the same DNA samples were used to generate either data set. The genotyping data were obtained using a custom microarray that resolves variant positions identified in the RefBeet sugar beet reference genome in comparison to other sugar beet lines[4]. Illumina sequences of the same set of accessions were used to calculate Mash distances. In order to evaluate the correlation between both distance matrices, we performed a Mantel test[3]. This test showed that the matrices were highly correlated, reflected by a correlation coefficient of r=0.7448. Furthermore, we generated individual phylogenetic trees with the same settings based on the two distance matrices of 255 accessions obtained from microarray-based genotyping and from Mash sketches, respectively. Although the distance matrices based on these two datasets had been computed in different ways, the two phylogenetic trees were very similar and the overall topology of the phylogenetic trees was identical. To assess the extent of similarity between the phylogenetic trees we calculated quartet distances[5] between them. In this method, the topology of all possible sets of four nodes is compared between trees. As a benchmark, we first determined the relatedness between two random trees consisting of 255 nodes, equivalent to 172 million

quartets. The random trees shared 57 million quartets, hence, displayed a quartet distance of 67%. In contrast, the comparison of the trees generated from microarray-based genotyping data and Mash sketches yielded 156 million equal quartets, resulting in a quartet distance of 12% indicating that their similarity is unlikely due to chance alone.

By adapting the settings for phylogenetic tree calculation we could further increase the similarity between the trees. The options “global rearrangements” (all possible placements of subtrees are tested) and multiple rounds of “jumbling” (entering accessions in random order) decreased the quartet distance to 9% (see Supplementary Table 1). As the two data sets were derived from two independent methods, we figured that the closer the two resulting phylogenetic trees were to each other, the more likely it is that the resulting topology is correct.

Supplementary Note 3. Sugar beet phylogeny details

In the tree comprising 290 *B. v. vulgaris* accessions and two outgroups (Fig. 5, Supplementary Fig. 5) the KWS subtree (orange branches) suggested that BETA 1375 was KWS material, and, in fact, it had been deposited by KWS at the IPK Gatersleben Genbank in 2003 under the name of KW-Zucca. For other accessions grouped into the KWS subtree relationships were less evident, e.g. PI 590615 is an old variety from Desprez and BETA 1333 corresponds to the old Monoricca variety from Hilleshög. Possibly, all these varieties share a common disease resistance donor which lead to their grouping together.

Closer inspection of the accessions to the right of the node marked in yellow revealed that four of these accessions (PI 285592 through PI 285595) had a description mentioning "crassa" (indicating fodder beet) in the USDA/GRIN database and that another one (PI 502293), labelled as "*Beta* sp.", had the information "fodder beet" in the Plant Inventory report that is linked to its passport data. These five accessions (brown bracket in Supplementary Fig. 5) clustered together close to the root of the tree. One accession (PI 357367, pale orange arrow) was called "cicla" (indicating leafy beets) in the database and another one (PI 222769, purple arrow) had the description "used as a vegetable" probably indicating either leaf beet or table beet. To study the relationship between beet cultitypes a larger number of accessions would be necessary.

The accessions to the left of the node marked in yellow (close to the node) seem not to be too far off from the accessions to the right of the "green" node in terms of breeding history, but they also seem disjointed, with a self-fertile type in there, C500 (PI 663878, marked by a blue arrow in Supplementary Fig. 5) which is an annual O-type tester (see passport data of the USDA GRIN database). Material from Fargo's breeding program (F1042 = PI 674103, exceptionally *Cercospora* sensitive), winter-hardy selections from Kiel, Germany (EL-A1200027), and NSCR (EL-A1402163), and some introgression lines between Western and Eastern US materials are among these accessions (blue circle).

The subtree to the left of the "green" node (more Western US-like) has a strong cluster of accessions that trace to Utah releases as a group (PI 610265, and PI 633945 - PI 633950; blue bracket), a group of narrowly related materials in a set of California genetic analysis lines for Fusarium resistance (EL-A1200035 through EL-A1200037; blue bracket), and a pair of sibling releases extracted from populations selected and evaluated for sugar beet cyst nematode resistance donated by germplasm contributions selected out of Salinas, California (PI 664914, PI 664916, PI 664922 and EL-A1600008; blue bracket). This subtree also encompasses the lines C869 (PI 628755, blue arrow) and US H20 (PI 631354, blue arrow), which is a very successful legacy hybrid whose seed parent is from Utah but whose pollen parent SP22 is Eastern US, here given as EL-A015030 (PI 615525, blue arrow). Both SF 'B' (EL-A1200026) and SF 'A' (EL-A029686), shaded in light blue, derive from populations related to the use of the self fertility allele donated by C869 (PI 628755).

In the Eastern US-enriched subtree to the right of the "green" node, there is a group of lines with breeding history that includes EL50/2 as a parent (PI 664912, highly tolerant to *Cercospora* leaf spot; blue arrow), e.g. lines EL-A1600024, EL-A027154, EL-A1600020, EL-A1402162, and EL-A1600025 (grey circle). Many others in this subtree seem to be products of the smooth-root (SR) phenotype introgression stemming from table beet back in the 1940's. Part of the goal was to return these SR types to adaption for Great Lakes growers, and SR98 (EL-A027149 = PI 655951, blue arrow) epitomizes that effort.

It is of interest that some lines from China are grouped together with breeding material from the USA (e.g. BETA 165, PI 518157, PI 518165; blue squares). These lines may be derivatives of accessions that were sourced from US materials, bred in China for a time, and then re-deposited in seed banks as new material.

Sugar beet accession SLC101 (PI 610268, blue arrow) is interesting as the donor of the monogermy trait that is used in every sugar beet hybrid in the world and, being self-fertile, contains low genetic diversity[6]. Another self-compatible sugar beet line is EL57 (PI 663212, blue arrow) which is a multigerm[7]. The two lines are quite different, but united around the self-fertility trait which may be the reason why they have been grouped together in our analysis.

The most distant sugar beet accession on the sugar beet phylogenetic tree is EL-A024961 (placement next to the root species, green arrow). This breeding line was derived from a screen of ca. 50 PI accessions of unadapted material for germination in 100 mM NaCl, a few germinated and were inter-crossed, planted, and selected in the field, and inter-crossed once more. The ones contributing to the EL-A024961 population were sugar beet accessions PI 232889, PI 355963, PI 518160, and PI 266100 (green arrows). Apparently, the addition and/or recombination of these disparate accessions caused a decline in similarity, resulting in the observed phylogenetic placement.

USDA germplasm enhancement efforts since the 1980's, including the East Lansing program, has increasingly focused on pollen parents adapted for each of the major beet growing areas in the US with an emphasis to broaden the genetic base of sugar beet[8]. Provenance can be ascribed to these locations, even with very low marker density[6], and revealed instances where shared germplasm is still recognizable in their genetic signatures (e.g. substantially derived accessions). Recent East Lansing pollen parents are generally derived through population improvement approaches, which results in slower fixation of favorable alleles than does strict inbreeding (e.g. higher residual heterozygosity). All of the East Lansing germplasm tested here is targeted to pollen parents, with primary selections for relative fecundity and field performance in test plots in Michigan, the Great Lakes growing region of the US. Operationally, seed was harvested from multi-line, inter-pollinated mother roots of two to 20 more-or-less diverse accessions with 5-40 individuals per accession, and the seed harvested was kept segregated by seed parent accession (these were often designated as 'mixing lines'). The bulk of East Lansing breeding material appears highly heterozygous[9]. Thus, genetic distance of East Lansing breeding populations is likely obfuscated by the breeding process as well as ascertainment uncertainties perhaps to a greater extent than wild accessions tested. Still, some known relationships from donor germplasm is consistent with their placement on the phylogenetic trees (e.g. four hitherto unsuspected related accessions from Utah, PI

66394x, and three from a narrow California base selected for Fusarium tolerance, EL-A120003x). It is interesting that each of these examples resolve to different higher-order subtrees. We assert that uniqueness of pollen parents is facilitated in large part by selection on generally oligo-genic traits originally derived from one or a few donor accessions, and genetic distance reflects the combination of various traits, wholly or partially, with respect to the accumulated genetic constitution of such traits. In this respect, tested accessions here have various targeted breeding goals such as *Cercospora* leaf spot resistance and Smooth Root and can be interpreted to occupy more closely related nodes in a manner consistent with their derivation. This information is useful to breeders, and finer discrimination could prove very useful for trait-gene discovery.

Supplementary Table 1. Comparison of tree calculation methods.

**Tree method**	**Quartets shared**	**Quartets differing**	**Distance**	**Computing time**
Random Topology	57100594	144960911	66.80%	n.a.
Neighbour Joining	135132004	36929501	21.50%	~5s
Fitch	151448152	20613353	12.00%	~1d
Fitch (Jumble 10x)	153857696	18203809	10.60%	~7d
Fitch (Jumble 3x, GR)	156213897	15847608	9.20%	~1M

Phylogenetic trees of a set of 255 beet accessions calculated from distances based on genotyping data and based on k-mer comparisons (Mash), respectively, were compared using quartet distances. “Random Topology” refers to a tree with arbitrarily arranged nodes. The Fitch algorithm provided the most reliable results, in particular when using both the “Jumble” option and “Global rearrangement” (GR), at the expense of an increase in computing time (single-core).

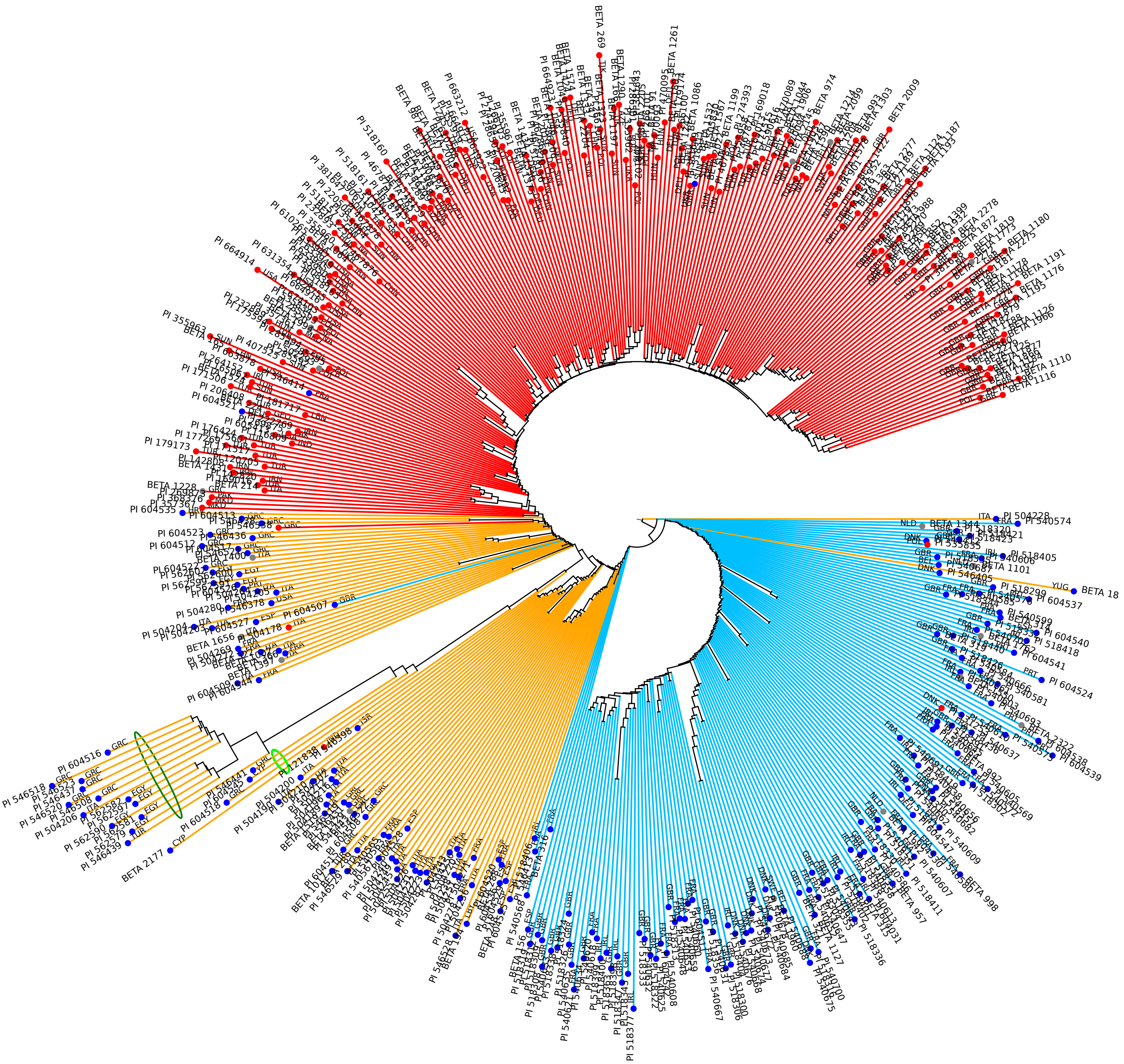

Supplementary Figure 1. Initial phylogenetic analysis of 474 sugar beet and sea beet accessions based on Mash distances revealed a group of 17 accessions labeled as sea beet (highlighted by green ellipses) that show a large distance to all other sea beets. The two outgroups were removed for better visibility. Leaf colors indicate taxonomic information prior to tree calculation (red: *B. v. vulgaris*, blue: *B. v. maritima*, grey: unknown subspecies, black: outgroup). Branch colors indicate *B. v. vulgaris* (red), sea beets from the Mediterranean area (yellow), and sea beets from Atlantic coasts (blue). Country codes were added according to information in public databases from USDA or IPK.

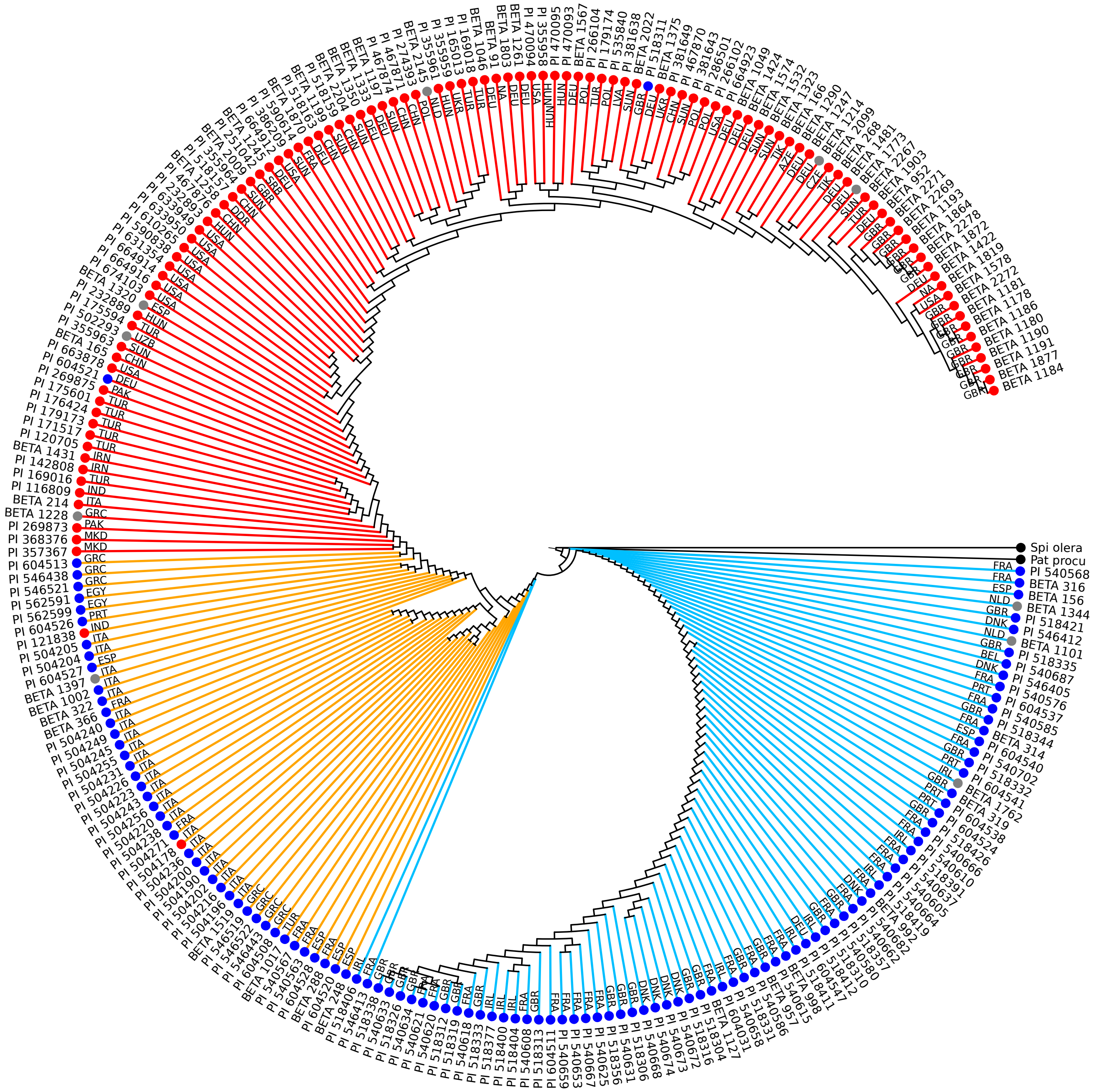

Supplementary Figure 2. Phylogenetic tree of sugar beet and sea beet accessions based on Mash distances. The 457 randomly sorted input accessions were divided in two subsets one of which is shown in this figure, the other one in Fig. 3. For comparability, 19 randomly selected accessions as well as the two outgroups spinach (Spi olera) and *Patellifolia procumbens* (Pat pro) are present in both trees. Tree calculation was performed with the Fitch algorithm based on pairwise Mash distances. Leaf colors indicate taxonomic information prior to tree calculation (red: *B. v. vulgaris*, blue: *B. v. maritima*, grey: unknown subspecies, black: outgroup). Branch colors indicate *B. v. vulgaris* (red), sea beets from the Mediterranean area (yellow), and sea beets from Atlantic coasts (blue). Country codes were added according to information in public databases form USDA or IPK.

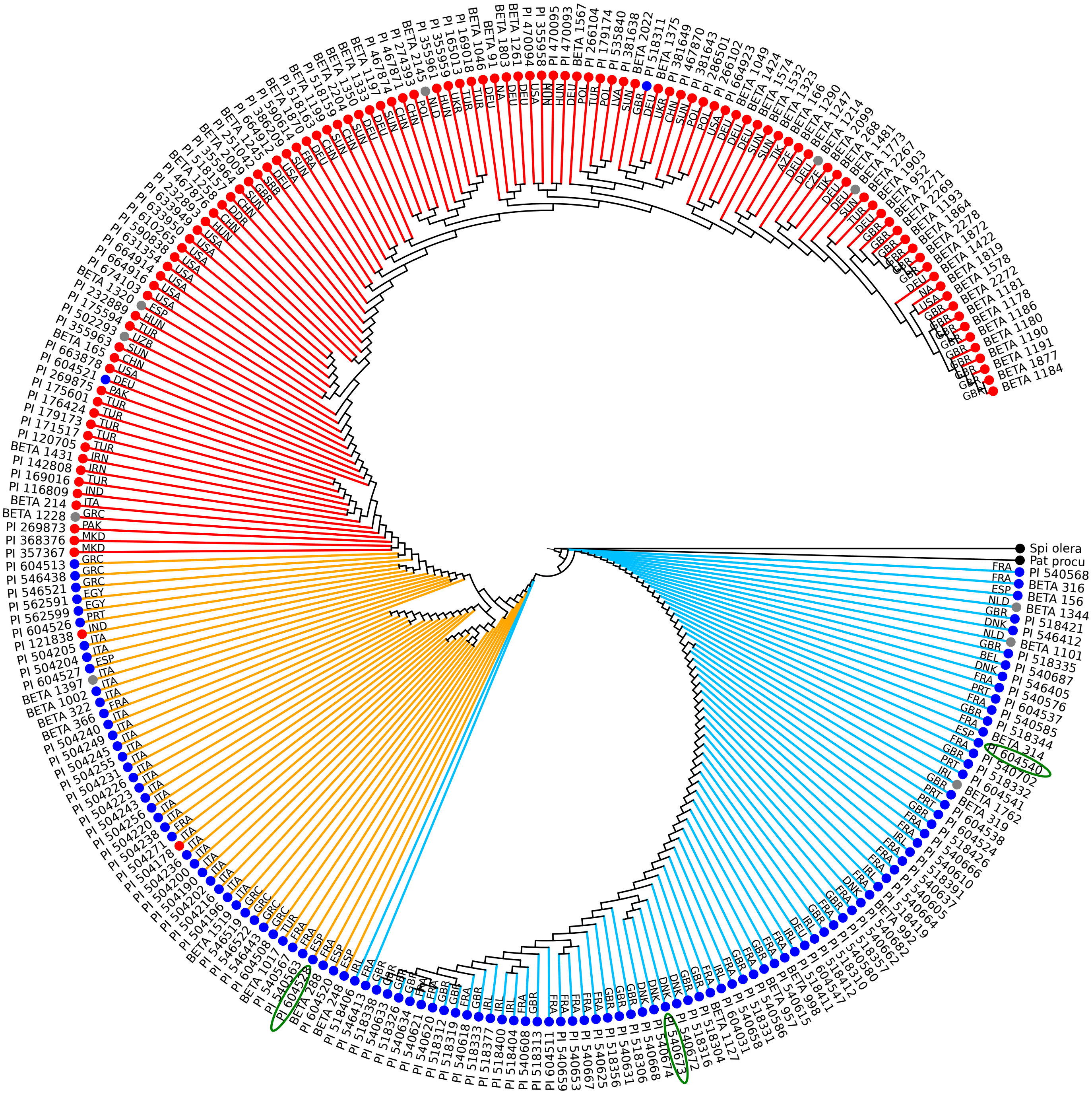

Supplementary Figure 3. Same as Supplementary Fig. 2 with accessions highlighted (green ellipses) that were mentioned in the text and were indicated by arrows on the map in Fig. 4.

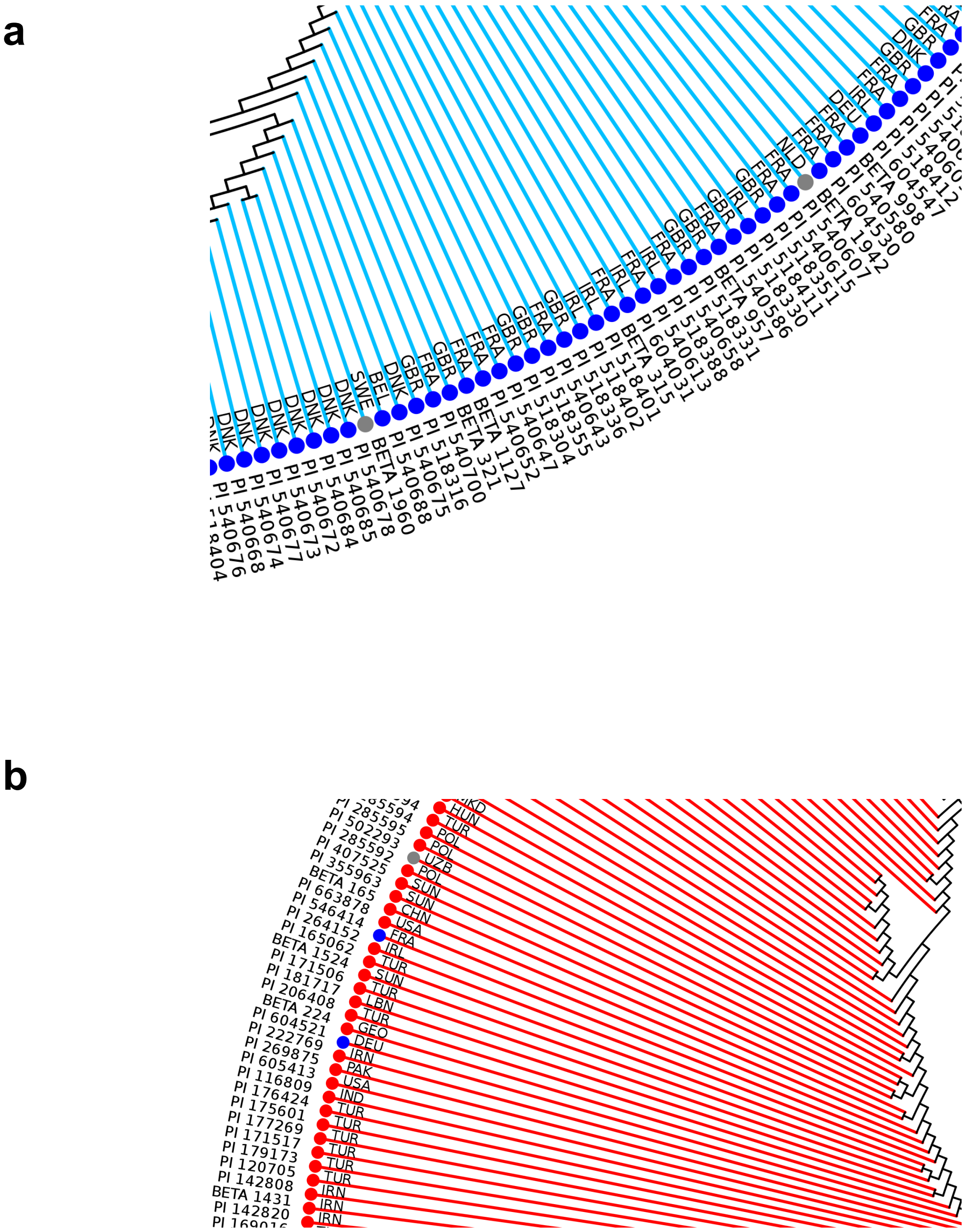

Supplementary Figure 4. Selection of the Atlantic sea beet subtree (a) and selection of the sugar beet subtree (b). The placement of *Beta* accessions of previously unknown subspecies assignment (grey circles) from Sweden (SWE) and The Netherlands (NLD) within the subtree of Atlantic sea beets provides evidence for their classification as *B. v. maritima* subspecies. In the sugar beet subtree, two accessions were labelled as subspecies *B. v. maritima* (blue circles), one accession was unassigned to any subspecies (grey circle). The placement of these three accessions within the sugar beet subtree suggests their classification as *B. v. vulgaris* subspecies.

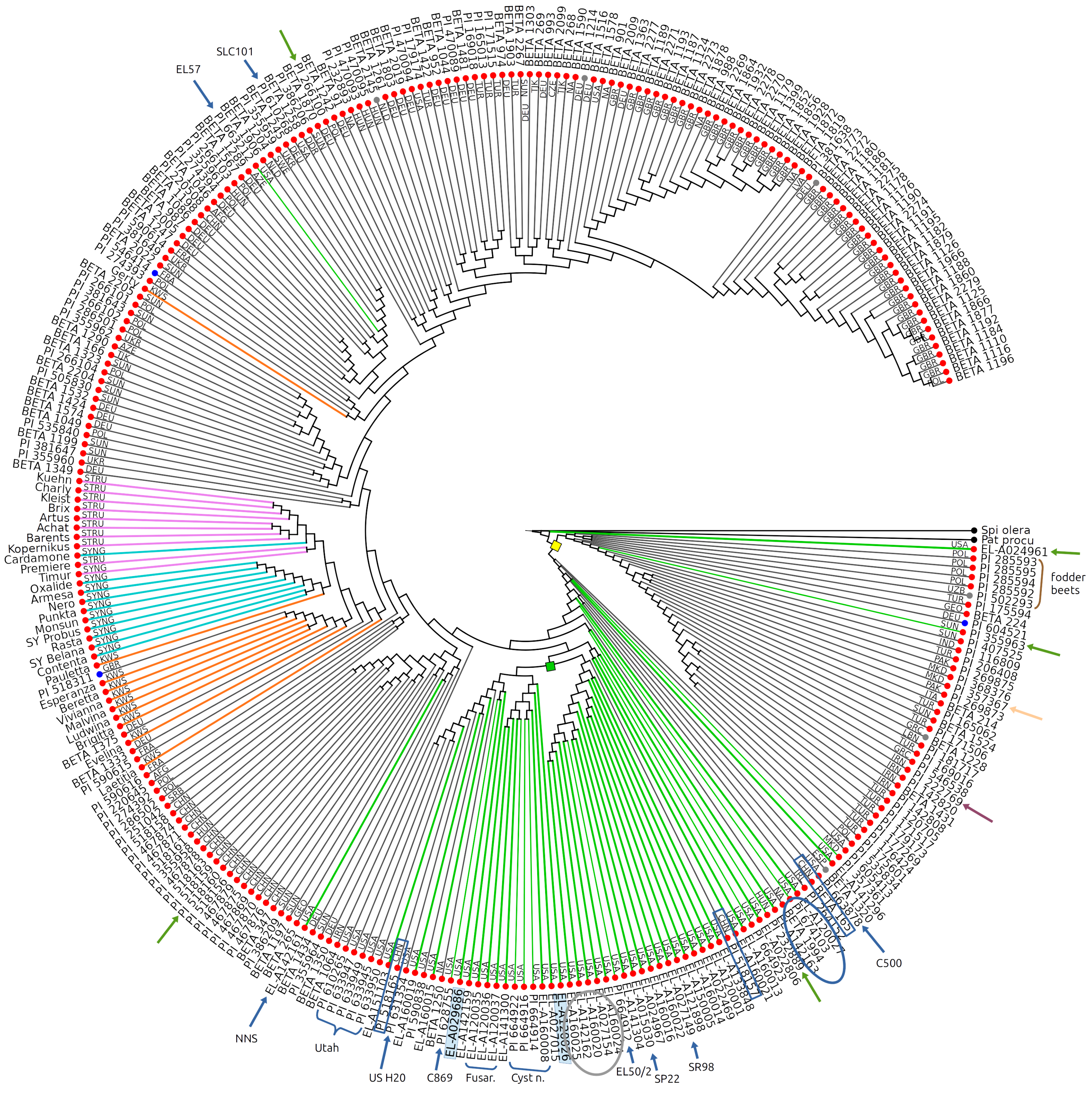

Supplementary Figure 5. Same as Fig. 5 but with accessions highlighted that are mentioned in the main text and in the Supplementary Notes.

Supplementary references

1. Stadermann, K. B., Weisshaar, B. & Holtgräwe, D. SMRT sequencing only de novo assembly of the sugar beet (Beta vulgaris) chloroplast genome. *BMC Bioinformatics* **16**, 295 (2015).
2. Kubo, T. *et al.* The complete nucleotide sequence of the mitochondrial genome of sugar beet (Beta vulgaris L.) reveals a novel gene for tRNACys(GCA). *Nucleic Acids Res.* **28**, 2571–2576 (2000).
3. Mantel, N. The detection of disease clustering and a generalized regression approach. *Cancer Res.* **27**, 209–220 (1967).
4. Dohm, J. C. *et al.* The genome of the recently domesticated crop plant sugar beet (Beta vulgaris). *Nature* **505**, 546–549 (2014).
5. Estabrook, G. F., McMorris, F. R. & Meacham, C. A. Comparison of Undirected Phylogenetic Trees Based on Subtrees of Four Evolutionary Units. *Syst. Biol.* **34**, 193–200 (1985).
6. McGrath, J. M., Derrico, C. A. & Yu, Y. Genetic diversity in selected, historical US sugarbeet germplasm and Beta vulgaris ssp. maritima: *Theor. Appl. Genet.* **98**, 968–976 (1999).
7. McGrath, J. M. Release of EL57 self-fertile sugarbeet germplasm. USDA-ARS Germplasm. *Release Not.* (2011).
8. Panella, L., Campbell, L. G., Eujayl, I. A., Lewellen, R. T. & McGrath, J. M. USDA-ARS Sugarbeet Releases and Breeding Over the Past 20 Years. *J. Sugarbeet Res.* **52**, (2015).
9. Galewski, P. & McGrath, J. M. Genetic diversity among cultivated beets (Beta vulgaris) assessed via population-based whole genome sequences. *BMC Genomics* **21**, 189 (2020).
