# Supplementary material for: Genomic distances reveal relationships of wild and cultivated beets
Source: Nat Commun. 2022 Apr 19;13:2021. doi: 10.1038/s41467-022-29676-9 (PMC9019029; doi:10.1038/s41467-022-29676-9)
Supplement: Supplementary file 2 — Description of Additional Supplementary Files [file 41467_2022_29676_MOESM2_ESM.pdf]

Description of Additional Supplementary Files

File Name: Supplementary Data 1
Description: Accessions of wild beets and sugar beets used for this study. Column designations: Accession: Accession identifier. Source: Accession source. The prefix BETA refers to accessions from IPK, prefix PI identifies accessions available from USDA (public seed repositories). KWS, STR, SYN represent the seed companies KWS SAAT SE, Strube Research GmbH, and Syngenta, respectively. ELA indicates the beet breeding program at East Lansing, Michigan, USA (there are three ELA accessions used in this study that subsequently received permanent PI identifiers: EL-A015030 = PI 615525, EL-A027149 = PI 655951, EL-A1501103 = PI 689015). Species: Taxonomic information available from passport data. Species proposed: Revised taxonomic assignment based on phylogenetic analysis. Latitude, Longitude: Geographical coordinates obtained from the USDA and IPK databases. Country: Country information for B. v. maritima and B. v. vulgaris accessions according to USDA and IPK databases. Coverage: Genomic coverage (conservatively estimated assuming a genome size of 758 Mbp) of Illumina whole-genome sequencing data after quality filtering as input for generating Mash sketches.
